# Supplementary material for: A Miniaturized Electrostatic Precipitator Respirator Effectively Removes Ambient SARS-CoV-2 Bioaerosols
Source: Viruses. 2022 Apr 6;14(4):765. doi: 10.3390/v14040765 (PMC9025737; doi:10.3390/v14040765)
Supplement: Supplementary file 1 [file viruses-14-00765-s001.zip › viruses-1591691-supplementary.pdf]

viruses

MDPI

Supplementary Materials

A Miniaturized Electrostatic Precipitator Respirator Effectively Removes Ambient SARS-CoV-2 Bioaerosols

Rachel K. Redmann [1,†], Brandon J. Beddingfield [1,†], Skye Spencer [1], Nicole R. Chirichella [1], Julian L. Henley [2,3], Wes Hager [4] and Chad J. Roy [1,5,*]

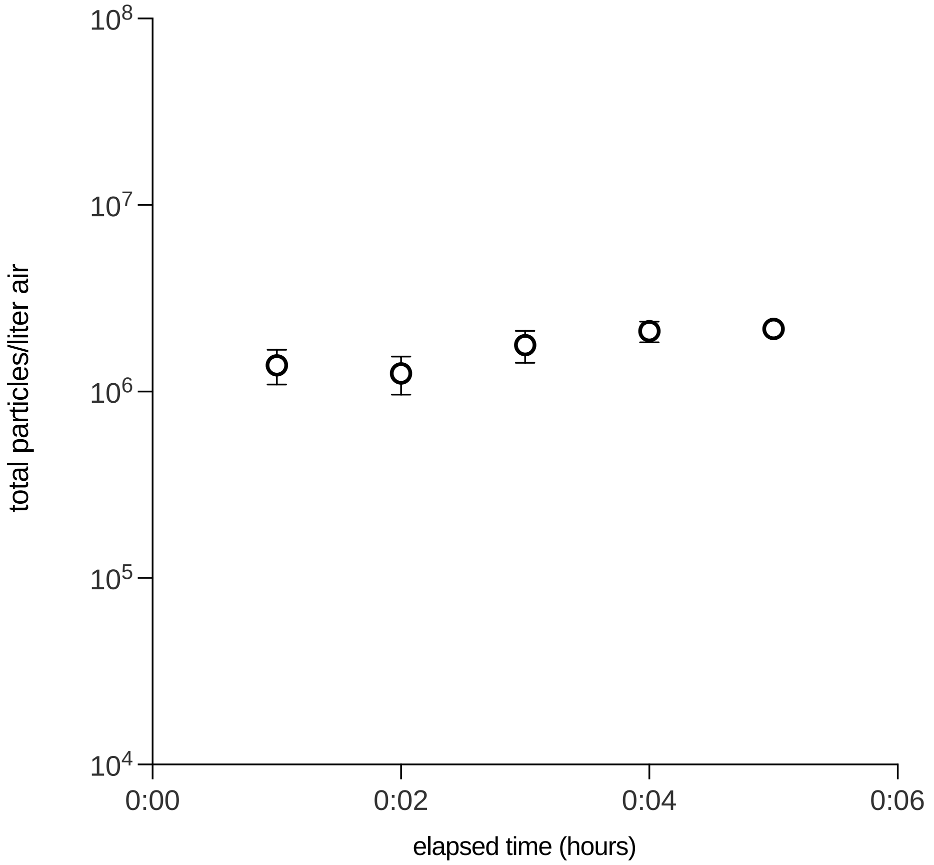

Figure S1. Testing system stability. Stability of the respirator exposure system as a function of total particles in the chamber environment on initiation (0:00) and throughout the exposure period. Open circles represent 60-sec average concentrations as measured by particle counter.

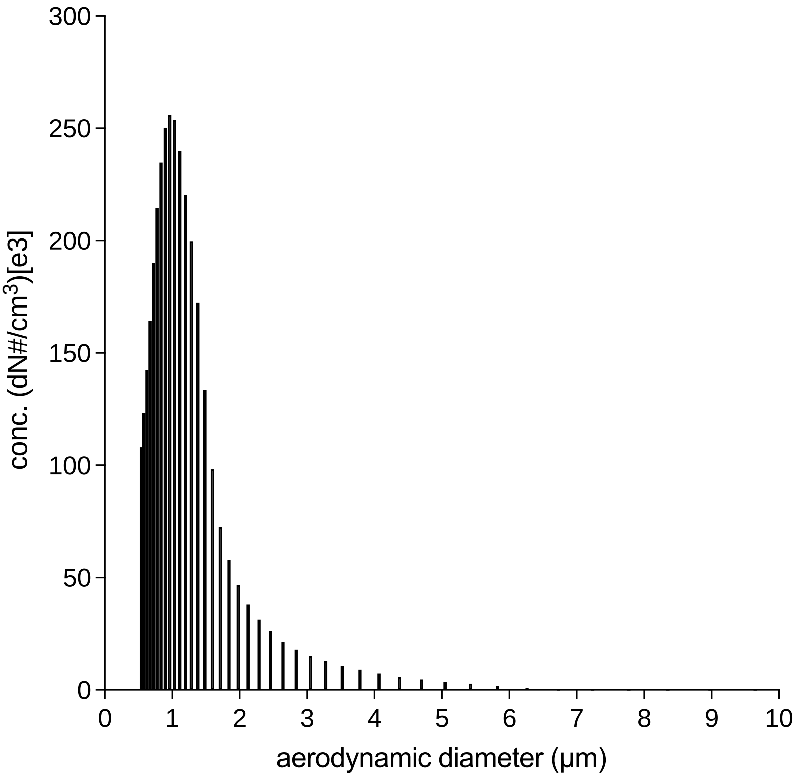

Figure S2. Representative particle size distribution in the respirator exposure chamber environment. Results indicated a MMAD of 1.3 µm and geometric standard deviation of 1.4 within the chamber.
